# Differential inflammation responses determine the variable phenotypes of epilepsy induced by *GABRG2* mutations

**DOI:** 10.1111/cns.14583

**Published:** 2024-02-15

**Authors:** Jiahui Sui, Longwu Zhan, Shengtao Ji, Wenwen Wu, Yuhan Chen, Feng Yun, Wenpeng Liang, Jie Wang, Maohong Cao, Dingding Shen, Qi Zhang

**Affiliations:** ^1^ Key Laboratory of Neuroregeneration of Jiangsu and Ministry of Education, Department of Neurology Affiliated Hospital of Nantong University, Medical School, Co‐innovation Center of Neuroregeneration, NMPA Key Laboratory for Research and Evaluation of Tissue Engineering Technology Products, Jiangsu Clinical Medicine Center of Tissue Engineering and Nerve Injury Repair, Nantong University Nantong China

**Keywords:** epilepsy, GABRG2, inflammatory response, mutation

## Abstract

**Objective:**

To explore the mechanism involved in variable phenotypes of epilepsy models induced by γ‐aminobutyric acid type A γ2 subunit (*GABRG2*) mutations.

**Methods:**

The zebrafish carrying wild‐type (WT) *GABRG2*, mutant *GABRG2(P282S)*, *GABRG2(F343L)* and *GABRG2(I107T)* were established by Tol2kit transgenesis system and Gateway method. Behavioral analysis of different transgenic zebrafish was performed with the DanioVision Video‐Track framework and the brain activity was analyzed by field potential recording with MD3000 Bio‐signal Acquisition and Processing System. The transcriptome analysis was applied to detect the underlying mechanisms of variable phenotypes caused by different *GABRG2* mutations.

**Results:**

The established Tg(*hGABRG2* ^*P282S*^) zebrafish showed hyperactivity and spontaneous seizures, which were more sensitive to chemical and physical epileptic stimulations. Traditional antiepileptic drugs, such as Clonazepam (CBZ) and valproic acid (VPA), could ameliorate the hyperactivity in *Tg(hGABRG2* ^*P282S*^ *)* zebrafish. The metabolic pathway was significantly changed in the brain transcriptome of *Tg(hGABRG2* ^*P282S*^ *)* zebrafish. In addition, the behavioral activity, production of pro‐inflammatory factors, and activation of the IL‐2 receptor signal pathway varied among the three mutant zebrafish lines.

**Conclusion:**

We successfully established transgenic zebrafish epileptic models expressing human mutant *GABRG2(P282S)*, in which CBZ and VPA showed antiepileptic effects. Differential inflammatory responses, especially the SOCS/JAK/STAT signaling pathway, might be related to the phenotypes of genetic epilepsy induced by *GABRG2* mutations. Further study will expand the pathological mechanisms of genetic epilepsies and provide a theoretical basis for searching for effective drug treatment.

## Graphical abstract

The transgenic zebrafish epileptic models expressing human mutant *GABRG2(P282S)* was established. Clonazepam (CBZ) and valproic acid (VPA) showed antiepileptic effects in the transgenic zebrafish. Differential inflammatory responses, especially SOCS/JAK/STAT signaling pathway, might be related with the phenotypes of genetic epilepsy induced by *GABRG2* mutations.

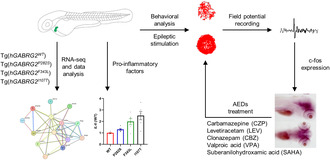

## 1 INTRODUCTION

Mutations in γ‐aminobutyric acid type A (GABA_A_) receptor subunit genes (*GABRs*) are frequently associated with various phenotypes of epilepsy, from mild epileptic syndromes such as childhood absence epilepsy (CAE) and febrile seizures (FS) to more severe epileptic phenotype such as Dravet syndrome (DS), Lennox–Gastaut syndrome (LGS), and West syndrome (WS).^1^, ^2^ GABA_A_ receptor is a chloride channel mediating most of the fast inhibitory neurotransmission in the central nervous system (CNS), which is usually composed of five subunits, two α1 subunits, two β2 subunits, and one γ2 subunit (2α12β2γ2). γ2 subunit (encoded by *GABRG2*) plays an important role in GABA_A_ receptor functions and mutations of γ2 subunit often induce the occurrence of epilepsy.^3^ Although some studies have investigated the relationship between genotypes and phenotypes of epilepsy induced by different *GABRG2* mutations,^4^ the mechanisms remain unclear.

The structure of γ2 subunit contains four transmembrane domains (TM1 to TM4) and a long extracellular N‐terminal domain. It was reported that the phenotype was more related to the location of variants within the protein and the variants in transmembrane domains had the most severe phenotype in terms of epilepsy.^4^ Our previous study has found mutations located in different structural domains leading to epileptic encephalopathies.^5^ To further investigate the complicated relationship between phenotypes and genotypes, we have established zebrafish epilepsy models overexpressing different mutant human *GABRG2*, including the F343L mutation located in the TM3^6^ and the I107T mutation located in the N‐terminal. These zebrafish models displayed spontaneous seizure activity and convulsive behaviors at the larval stage. Compared with rodent models, the shorter maturation period of zebrafish provides a better platform for investigating the mechanisms of epilepsy with different genotypes and screening of antiepileptic drugs (AEDs). The establishment of the above zebrafish epilepsy models with GABRG2 mutations facilitated the study of molecular mechanisms involved in the relationship between genotypes and phenotypes of epilepsy, thus promoting the development and clinical application of specific AEDs.

Previous research has indicated that neuroinflammation is involved in epileptogenesis.^7^ We have also demonstrated that neuroinflammation was induced in febrile seizures, and the increased pro‐inflammatory factors, such as interleukin 1β (IL‐1β) and IL‐6, might be responsible for the epileptogenesis in fever‐associated epilepsy.^8^ Increased pro‐inflammatory cytokines including tumor necrosis factor‐alpha (TNF‐α), IL‐1β, and IL‐6 were also observed in an epilepsy mouse model with Gabrg2^+/Q390X^ knockin, indicating neuroinflammation was one of the mechanisms for genetic epilepsy.^9^ However, whether neuroinflammation is involved in other phenotypes of epilepsy induced by *GABRG2* mutations and is related to the severity of epilepsy needs further exploration.

In this study, we first established a new transgenic zebrafish line carrying mutant human *GABRG2(P282S)*, which occurred in the TM1 and observed its epileptic phenotype. Then we tested some traditional and new AEDs in this epilepsy model to find out effective AEDs. In addition, we compared the behavior characters among three different mutant GABRG2 transgenic zebrafish as well as the transcriptome changes to further understand the underlying mechanisms involved in variable phenotypes induced by different *GABRG2* mutations.

## 2 METHODS

### 2.1 Zebrafish establishment and maintenance

The zebrafish carrying wild‐type (WT) *GABRG2*, mutant *GABRG2(P282S)*, *GABRG2(F343L)* and *GABRG2(I107T)*, referred to as Tg(*hGABRG2* ^*WT*^), Tg(*hGABRG2* ^*P282S*^), Tg(*hGABRG2* ^*F343L*^) and Tg(*hGABRG2* ^*I107T*^), respectively, were established with Tol2kit transgenesis system and Gateway vectors according to our previous protocols^6^ and maintained at 28°C in a 14 h light/10 h dark cycle with a circulating water system (pH at 7.0–8.0) in the Zebrafish Center of Nantong University under conditions. All protocols and procedures followed the institutional animal care guidelines of Nantong University (20200305–001). The embryos and larvae were cultured in the E3 medium (5.0 mM NaCl, 0.17 mM KCl, 0.33 mM MgSO_4_, 0.33 mM CaCl_2_) with 0.2 mM 1‐phenyl‐2‐thio‐urea (PTU) added at 24 h post‐fertilization (hpf) to inhibit pigmentation.

### 2.2 Behavioral analysis

The swimming movements of the zebrafish larvae at 5 days post‐fertilization (dpf) were recorded with the DanioVision Video‐Track framework (Noldus, German) for 30 min after 30 min adaption in the recording chamber. The zebrafish induced with 15 mM PTZ were also recorded for 30 min right after the stimulation. The zebrafish stimulated with light flash stimulation (light on for 5 s and turn off for 5 s) were recorded for a total of 100 cycles. All the data were analyzed with the EthoVision XT locomotion tracking software (Noldus, Germany). The average distance traveled, the activity of mobility, and the time spent in movement for each group were calculated and compared.

### 2.3 Whole‐mount in situ hybridization (WISH) and the quantitative reverse transcription polymerase chain reaction (qRT‐PCR)

The mRNA expressions of *c‐fos* in the brains of zebrafish were measured with WISH and qRT‐PCR. The WISH was performed with the modified Thisse's method as previously reported.^6^ qRT‐PCR was performed with SYBR green mix (Vazyme, China) after RNA extraction with TRIzol reagent (Invitrogen, USA) and cDNA transcription with an Omniscript RT kit (Qiagen, USA) according to the manufacturer's protocols. The sequences of primers used are listed in Table S1. *β‐actin* was used as the internal control. Data were normalized to the internal reference gene *β‐actin* and quantified relative to the expressions in the WT control larvae. Values represented averages from several independent biological samples as indicated in the figure legends and each sample included 10 pooled larvae.

### 2.4 Field potential recording

The field potential recording of zebrafish was performed with the MD3000 Bio‐signal Acquisition and Processing System (Anhui Zhenghua, China). In brief, the zebrafish were embedded in recording media solution (1 mM NaCl, 2.9 mM KCl, 10 mM HEPES, 1.2 mM MgCl_2_, 10 mM Dextrose, 2.1 mM CaCl_2_) with 1.2% low melting agarose and 0.02% tricaine to anesthetize the animals. The field potential recording was performed for each zebrafish with electrodes inserted into the forebrain. The diaphragm clamp amplifier was set to a magnification of 1000, an upper limit of 10,000 Hz, a time constant of 0.5 ms, and a high‐pass filter of 100 Hz.

### 2.5 Drug treatment

At 5 dpf, the Tg(*hGABRG2* ^*P282S*^) zebrafish were treated with different antiepileptic drugs (AEDs) for 30 min followed by locomotor activity recording for 30 min. All the drugs (Sigma‐Aldrich) were dissolved in an E3 medium with dimethylsulfoxide (DMSO; Sigma‐Aldrich). The working concentration of each drug was 100 μM for carbamazepine (CZP), 30 mM for levetiracetam (LEV), 100 μM for clonazepam (CBZ), 50 μM for valproic acid (VPA) and 2.5 μM for suberanilohydroxamic acid (SAHA, also known as Vorinostat). To further confirm the effects of CBZ and VPA, 25 μM, 50 μM, and 100 μM of CBZ and VPA were applied.

### 2.6 Transcriptomic assay

The brain samples underwent RNA extraction using TRIzol Reagent and 1 μg total RNA with a RIN value above 6.5 was used for the following library preparation and transcriptome sequencing using the HiSeq Control Software (HCS) + OLB + GAPipeline‐1.6 (Illumina) on the HiSeq instrument. Gene sets with a *p* < 0.05 and a false discovery rate <0.05 were considered significantly enriched genes. Gene ontology (GO) and Kyoto Encyclopedia of Genes and Genomes (KEGG) pathway analysis were conducted as previously reported.^10^ The protein–protein interaction (PPI) network of the differentially expressed genes (DEGs) was established with the STRING database (https://cn.string‐db.org/). The data analysis was processed by GENEWIZ (Suzhou, China).

### 2.7 Statistical analyses

Data are expressed as mean ± SEM. Statistical analysis was performed using GraphPad Prism 8.0 (GraphPad, San Diego, CA, USA). The normality of all data was analyzed by Kolmogorov–Smirnov test or Shapiro–Wilk test. Unpaired Student's *t*‐test or one‐way ANOVA and subsequent Tukey's multiple comparisons test were used to compare the differences between groups when the data exhibited normal distribution. Mann–Whitney *U*‐test or Kruskal–Wallis test and subsequent Dunn's multiple comparisons test were used when the data did not conform to a normal distribution. Statistically significant was set as *p* < 0.05.

## 3 RESULTS

### 3.1 The establishment of Tg( *hGABRG2* ^*P282S*^ ) zebrafish with hyperactivity and spontaneous seizures

To generate transgenic zebrafish with neuronal specifically expressing WT *GABRG2* or mutant *GABRG2*, we used pDestTol2CG2 plasmid with neuronal‐specific *HuC* promoter (Figure S1A,B). The cmlc2: EGFP in the vector was to label the embryonic heart with enhanced green fluorescent protein (EGFP) for screening embryos with human *GABRG2* expression. The recombinant plasmids were microinjected into one‐cell stage zebrafish embryos with transposase mRNA and the embryos with EGFP‐labeled hearts were screened out for further breeding (Figure S1C). The expression of human *GABRG2* and the genomic sequence of transgenic zebrafish were confirmed with RT‐PCR (Figure S1D) and Sanger sequencing (Figure S1E), showing the change of “C” to “T” at 844, which would translate from Proline (P) to Serine (S) at site 282 in the mutant group. The above results suggested that we successfully established transgenic zebrafish carrying human WT *GABRG2* or mutant *GABRG2(P282S)*.

**FIGURE 1:**
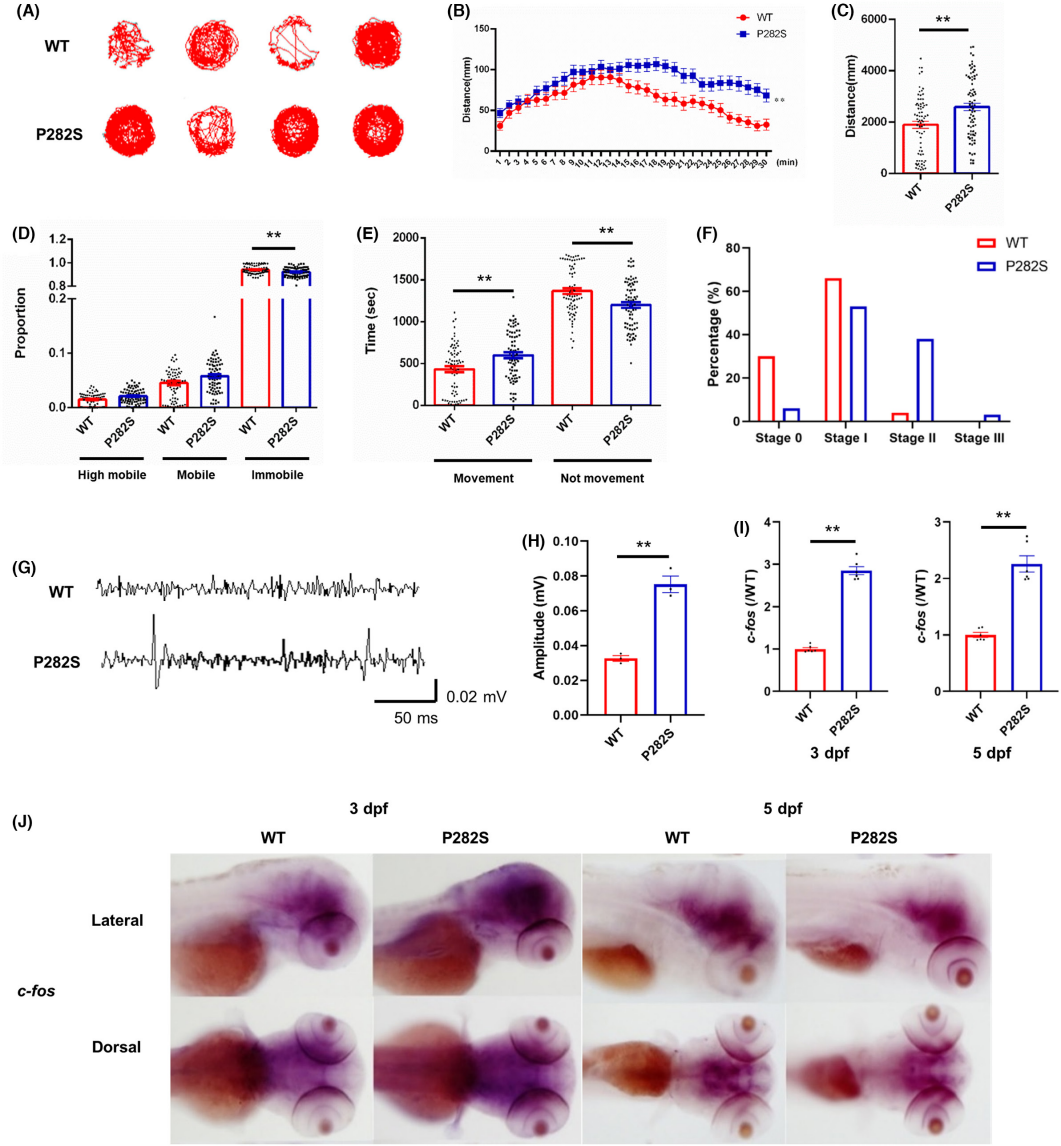
The establishment of Tg(*hGABRG2* ^*P282S*^) zebrafish with hyperactivity and spontaneous seizures. (A) The representative locomotor activity of WT and P282S transgenic zebrafish larvae at 5 dpf recorded in the DanioVision Video‐Track system with EthoVision XT locomotion tracking software. (B) The distance of the transgenic zebrafish traveled every minute during the 30‐min recording time. (C) The quantification of the total distance traveled for each group. **p < 0.01 by Mann–Whitney *U*‐test (*n* = 73 in WT and *n* = 75 in P282S group). (D) The activity mobility of the transgenic zebrafish larvae was defined as immobile (proportion of activity time < 20%), mobile (proportion of activity time between 20% and 60%), and highly mobile (proportion of activity time > 60%). *p < 0.05, **p < 0.01 by one‐way ANOVA and subsequent Tukey's multiple comparisons (*n* = 73 in WT and *n* = 75 in P282S group). (E) The quantification of time spent in movement or not movement of the transgenic zebrafish larvae. **p < 0.01 by one‐way ANOVA and subsequent Tukey's multiple comparisons (*n* = 73 in WT and *n* = 75 in P282S group). (F) The percentage of WT and P282S zebrafish larvae reaching stage 0 to stage III at 5 dpf (*n* = 73 for WT larvae and *n* = 75 for P282S larvae). (G) The representative field recording was obtained from the WT and P282S transgenic zebrafish larvae. (H) The quantification of maximal amplitude of EEG. **p < 0.01 by Student's *t*‐test (*n* = 3 for each group). (I) Quantification of *c‐fos* mRNA expressions in WT and P282S zebrafish larvae at 3 dpf and 5 dpf by qRT‐PCR. The expression level in the WT group was set as “1” after normalization to the internal reference gene *β‐actin*. **p < 0.01 by Student's *t*‐test (*n* = 6 for each group and one sample = 10 pooled larvae). (J) WISH for *c‐fos* expression (dark purple) within the whole brain of WT and P282S larval zebrafish at 3 dpf and 5 dpf.

Locomotor activities monitored with a video tracking system (Noldus, German) were used to investigate whether the expression of mutant *GABRG2(P282S)* would affect the neuronal hyperexcitability of the transgenic zebrafish. At 5 dpf, the representative swimming tracks of Tg(*hGABRG2* ^*WT*^) and Tg(*hGABRG2* ^*P282S*^) larvae were shown in Figure 1A. The distance traveled in the 30‐min recording was plotted in Figure 1B and the total distance traveled in each group was quantified in Figure 1C. The swimming distance increased significantly in the P282S group (2592 ± 140 mm vs. 1899 ± 134 mm in the WT group, *p* = 0.0013). The proportion of P282S zebrafish in immobile activity decreased significantly (92.10 ± 0.46% in the P282S group vs. 93.96 ± 0.49% in WT group, *p* = 0.0033, Figure 1D) while the movement duration increased in the P282S zebrafish (599.6 ± 34.00 s vs. 432.3 ± 34.55 s in WT group, *p* = 0.0036, Figure 1E).

According to the epilepsy scoring system of zebrafish larvae, the activities are divided into four stages: Stage 0 (little swimming activity); Stage I (a general increase in swimming activity); Stage II (rapid “whirlpool‐like” swimming behavior); Stage III (convulsions and loss of posture).^11^ At 5 dpf, about 30% and 66% of the WT zebrafish stayed in Stage 0 and Stage I, respectively, while only 4% reached Stage II and no zebrafish reached Stage III. On the other hand, only 6% of P282S zebrafish stayed in Stage 0. The proportion of mutant P282S zebrafish reaching Stage I (53%) and Stage II (38%) increased significantly. In addition, 3% of zebrafish reached Stage III in the P282S group (Figure 1F).

Field potential recording in the forebrain was performed to observe the seizure activity in the zebrafish larvae. Multispike, large‐amplitude, and long‐duration burst discharges were detected in the P282S zebrafish (Figure 1G), with a significant increase of the maximal amplitude (0.075 ± 0.005 vs. 0.033 ± 0.002, *p* = 0.0011, Figure 1H). To further detect the brain activity in transgenic zebrafish, the transcription factor, *c‐fos*, a hallmark of seizure onset in vertebrates, was measured with WISH and qRT‐PCR. The qRT‐PCR quantification analysis showed that the expression of *c‐fos* mRNA in the P282S group was much higher than that in the WT control (3 dpf: 2.850 ± 0.096 vs. 1.000 ± 0.033, *p* < 0.0001; 5 dpf: 2.258 ± 0.142 vs. 1.000 ± 0.043, *p* < 0.0001, Figure 1I). WISH images showed that *c‐fos* was expressed throughout the brain, including telencephalon, optic tectum, midbrain, and hindbrain at 3 and 5 dpf (Figure 1J). The above results suggested that P282S zebrafish was more active in the swimming behavior and had spontaneous seizures at the larval stage, indicating a successful establishment of a zebrafish epilepsy model carrying human mutant *GABRG2 (P282S)*.

### 3.2 The locomotor activity changed with PTZ exposure and light flash stimulation

**FIGURE 2:**
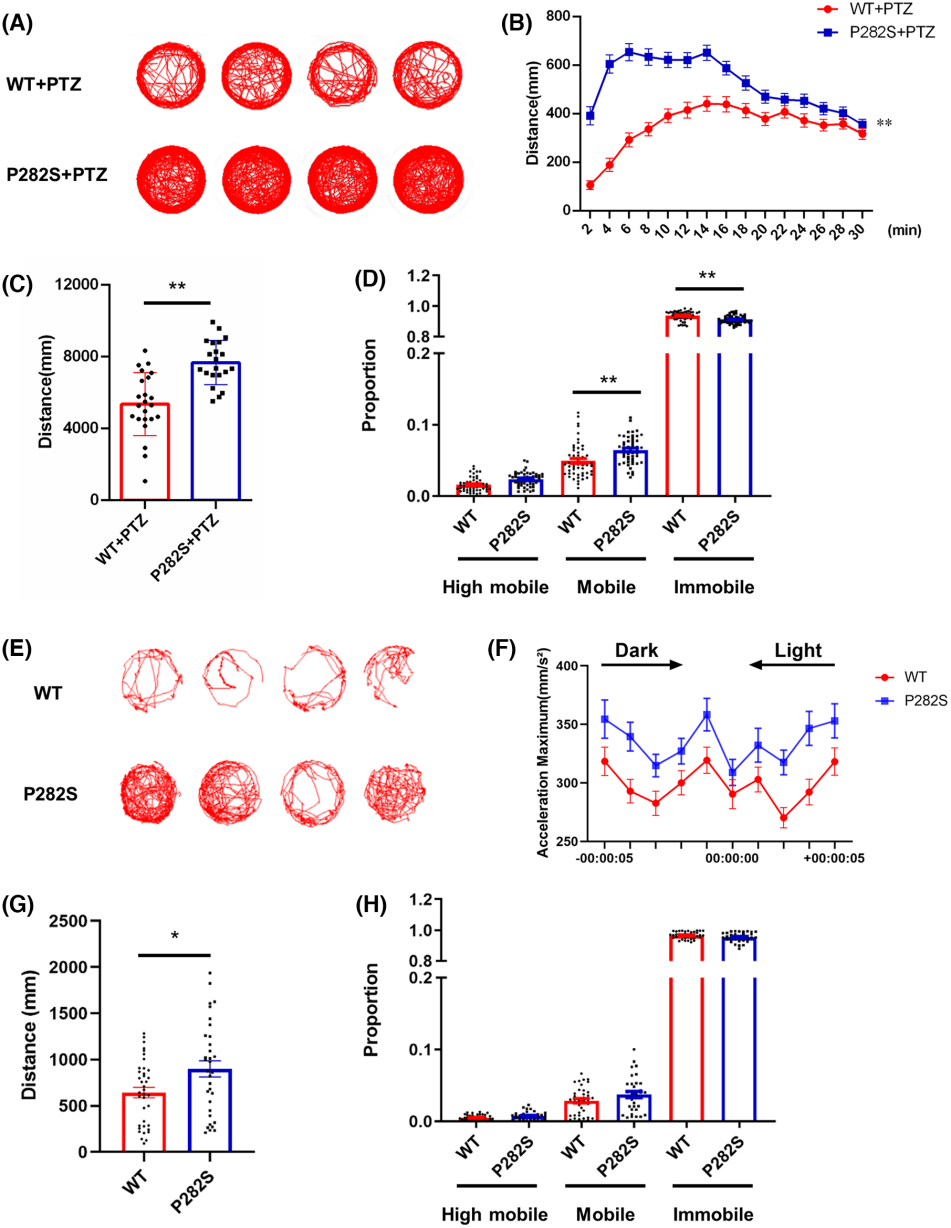
The locomotor activity changes of Tg(*hGABRG2* ^*P282S*^) zebrafish with PTZ exposure and light flash stimulation. (A) The representative locomotor activity of WT and P282S transgenic zebrafish larvae at 5 dpf with 15 mM PTZ exposure. (B) The diagram showing the distance of the transgenic zebrafish traveled during the 30‐min recording time after PTZ exposure. (C) The quantification of the total distance traveled for each group with PTZ exposure. **p < 0.01 by Student's *t*‐test (*n* = 23 in WT and *n* = 21 in P282S group). (D) The mobility of the transgenic zebrafish larvae with PTZ exposure. **p < 0.01 by one‐way ANOVA and subsequent Tukey's multiple comparisons (*n* = 23 in WT and *n* = 21 in P282S group). (E) The representative locomotor activity of WT and P282S transgenic zebrafish larvae measured on 5 dpf during light flash stimulation (light on for 5 s and off for 5 s, a total of 100 cycles). (F) The diagram showing the acceleration maximum of the transgenic zebrafish during the light flash stimulation. (G) The quantification of the total distance traveled for each group with light flash stimulation. *p < 0.05 by Student's *t*‐test (*n* = 38 in WT and *n* = 33 in P282S group). (H) The mobility of the transgenic zebrafish larvae with light flash stimulation (*n* = 38 in WT and *n* = 33 in the P282S group).

PTZ is an antagonist of GABA_A_ receptors which is often used to induce seizures in rodents and zebrafish. The swimming behavior trajectory was monitored for 30 min after 15 mM PTZ exposure at 5 dpf (Figure 2A,B). The moving distance of P282S mutant zebrafish increased significantly (7673 ± 270 mm vs. 5357 ± 364 mm, *p* < 0.0001, Figure 2C). The proportion of P282S zebrafish in immobile activity decreased significantly (91.18 ± 0.36% vs. 93.70 ± 0.41% in WT group, *p* < 0.0001), while zebrafish in mobile and high mobile activity increased (mobile: 6.46 ± 0.27% in P282S group vs. 4.92 ± 0.33% in WT group, *p* = 0.0040; high mobile: 2.37 ± 0.13% in P282S group vs. 1.57 ± 0.14% in WT group, *p* = 0.4100, Figure 2D).

Frequent light flash stimulation is inducement of seizures in some patients with epilepsy. To detect whether the mutant P282S zebrafish also has a similar phenotype, we set the light flash stimulus condition with 100 cycles of 5 s light on and 5 s light off. The swimming trajectory is plotted in Figure 2E. Upon light stimulation, we noticed that both the acceleration maximum (Figure 2F) and total distance traveled in P282S larvae increased (898.8 ± 87.06 mm vs. 643.6 ± 56.15 mm in the WT group, *p* = 0.0138, Figure 2G), indicating a hyperactive response to light flash exposure. However, the mobile activity of P282S larvae was not significantly changed with light flash stimulation (Figure 2H). These results suggested that the P282S mutant zebrafish were more sensitive to chemical and physical stimulations which would induce epileptogenesis.

### 3.3 CBZ and VPA ameliorated hyperactivity in *Tg(* *hGABRG2* ^*P282S*^ *)* zebrafish

**FIGURE 3:**
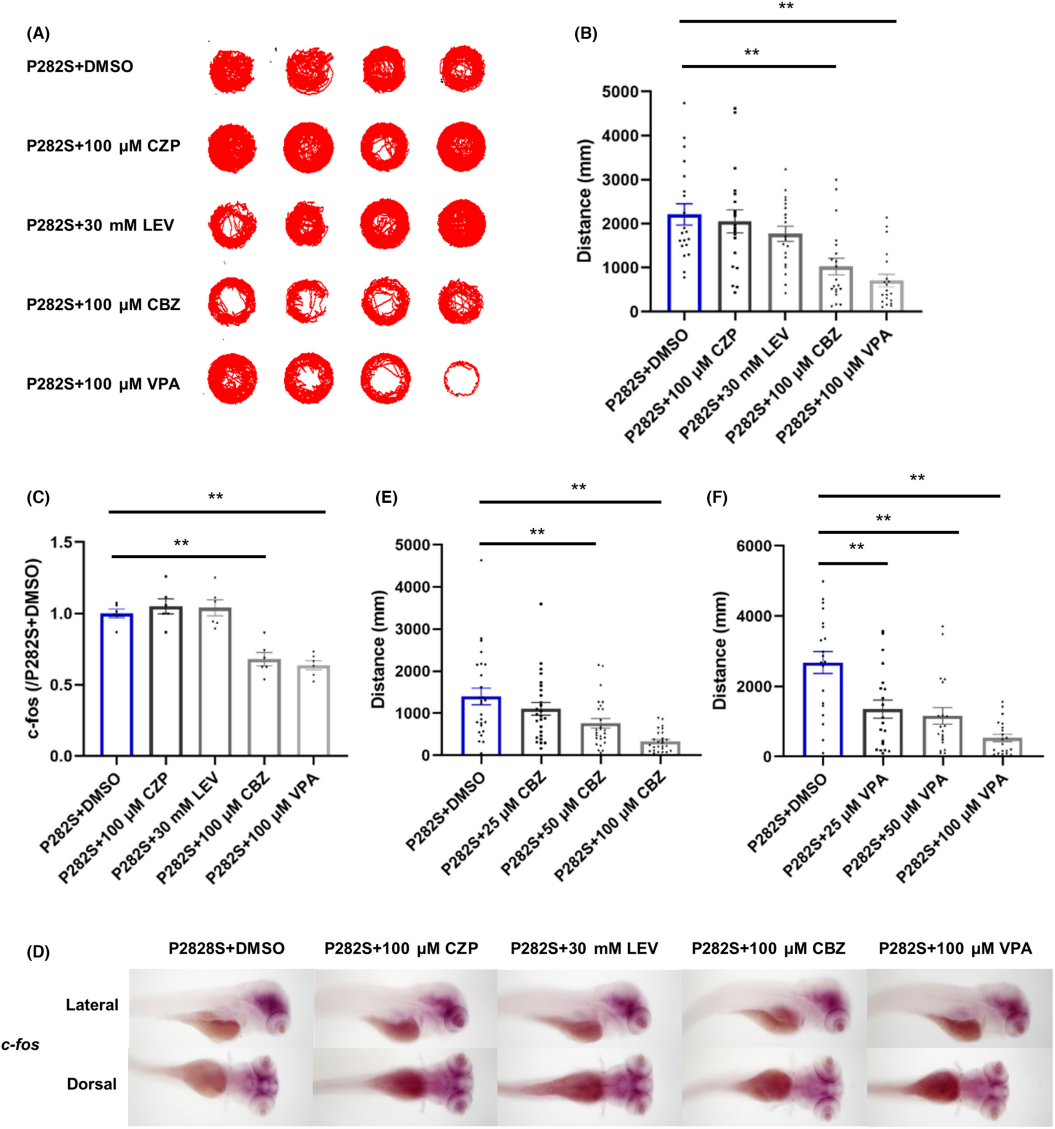
The effects of different traditional AEDs in Tg(*hGABRG2* ^*P282S*^) zebrafish. (A) The representative locomotor activity of P282S transgenic zebrafish larvae treated with different traditional AEDs (100 μM CZP, 30 mM LEV, 100 μM CBZ, 100 μM VPA) for 30 min at 5 dpf. (B) The total distance of the P282S transgenic zebrafish larvae traveled with different traditional AEDs treatments. **p < 0.01 by Kruskal–Wallis test and subsequent Dunn's multiple comparisons test (*n* = 20 in each group). (C) Quantification of *c‐fos* mRNA expressions in the P282S zebrafish larvae treated with different traditional AEDs at 5 dpf by qRT‐PCR. The expression level in the P282S + DMSO group was set as “1” after normalization to the internal reference gene *β‐actin*. **p < 0.01 by one‐way ANOVA and subsequent Tukey's multiple comparisons (*n* = 6 for each group and one sample = 10 pooled larvae). (D) WISH for *c‐fos* expression (dark purple) within the whole brain of P282S larval zebrafish treated with different traditional AEDs (100 μM CZP, 30 mM LEV, 100 μM CBZ, 100 μM VPA) for 30 min at 5 dpf. (E) The quantification of the total distance traveled with different concentrations (25, 50, 100 μM) of CBZ. **p < 0.01 by one‐way ANOVA and subsequent Tukey's multiple comparisons (*n* = 26 in each group). (F) The quantification of the total distance traveled with different concentrations (25, 50, 100 μM) of VPA. **p < 0.01 by one‐way ANOVA and subsequent Tukey's multiple comparisons (*n* = 20 in each group).

As the patient carrying P282S mutation showed secondary generalized onset and remained intractable with slight improvement with lamotrigine (LTG) treatment, we screened some traditional AEDs in the *Tg*(*hGABRG2* ^*P282S*^) zebrafish model to give a hint for further clinical application. Various drugs, including 100 μM CZP, 30 mM LEV, 100 μM CBZ, and 100 μM VPA, were added to the culture medium for 30 min at 5 dpf and the locomotor activity was recorded (Figure 3A). The total distance in each group was calculated and the data indicated that CBZ and VPA could significantly decrease the swimming distance in the P282S zebrafish (DMSO: 2210 ± 242.9 mm; CZP: 2053 ± 258.3 mm, *p* = 0.9313; LEV: 1771 ± 172.5 mm, *p* = 0.9684; CBZ: 1024 ± 189.2 mm, *p* = 0.0003; VPA: 705.3 ± 141.0 mm, *p* < 0.0001; Figure 3B). In addition, the decreased *c‐fos* expression in the P282S zebrafish brain also suggested less hyperactivity with CBZ and VPA treatment (DMSO: 1.000 ± 0.030; CZP: 1.050 ± 0.053, *p* > 0.9999; LEV: 1.040 ± 0.056, *p* > 0.9999; CBZ: 0.680 ± 0.047, *p* = 0.0047; VPA: 0.637 ± 0.032, *p* < 0.0001; Figure 3C,D), indicating that CBZ and VPA might display an antiepileptic effect in the *Tg*(*hGABRG2* ^*P282S*^) zebrafish. Then we used different concentrations of CBZ and VPA to further confirm their effects. Both CBZ (DMSO: 1397 ± 199.2 mm; 25 μM: 1104 ± 151.4 mm, *p* = 0.4526; 50 μM: 759.1 ± 113.0 mm, *p* = 0.0089; 100 μM: 328.7 ± 48.73 mm, *p* < 0.0001; Figure 3E) and VPA (DMSO: 2679 ± 313.7 mm; 25 μM: 1355 ± 257.9 mm, *p* = 0.0012; 50 μM: 1160 ± 238.0 mm, *p* = 0.0002; 100 μM: 530.6 ± 107.3 mm, *p* < 0.0001; Figure 3F) showed a concentration‐dependent effect on the swimming distance of P282S zebrafish.

### 3.4 Comparison of different mutant *GABRG2* transgenic zebrafish models

**FIGURE 4:**
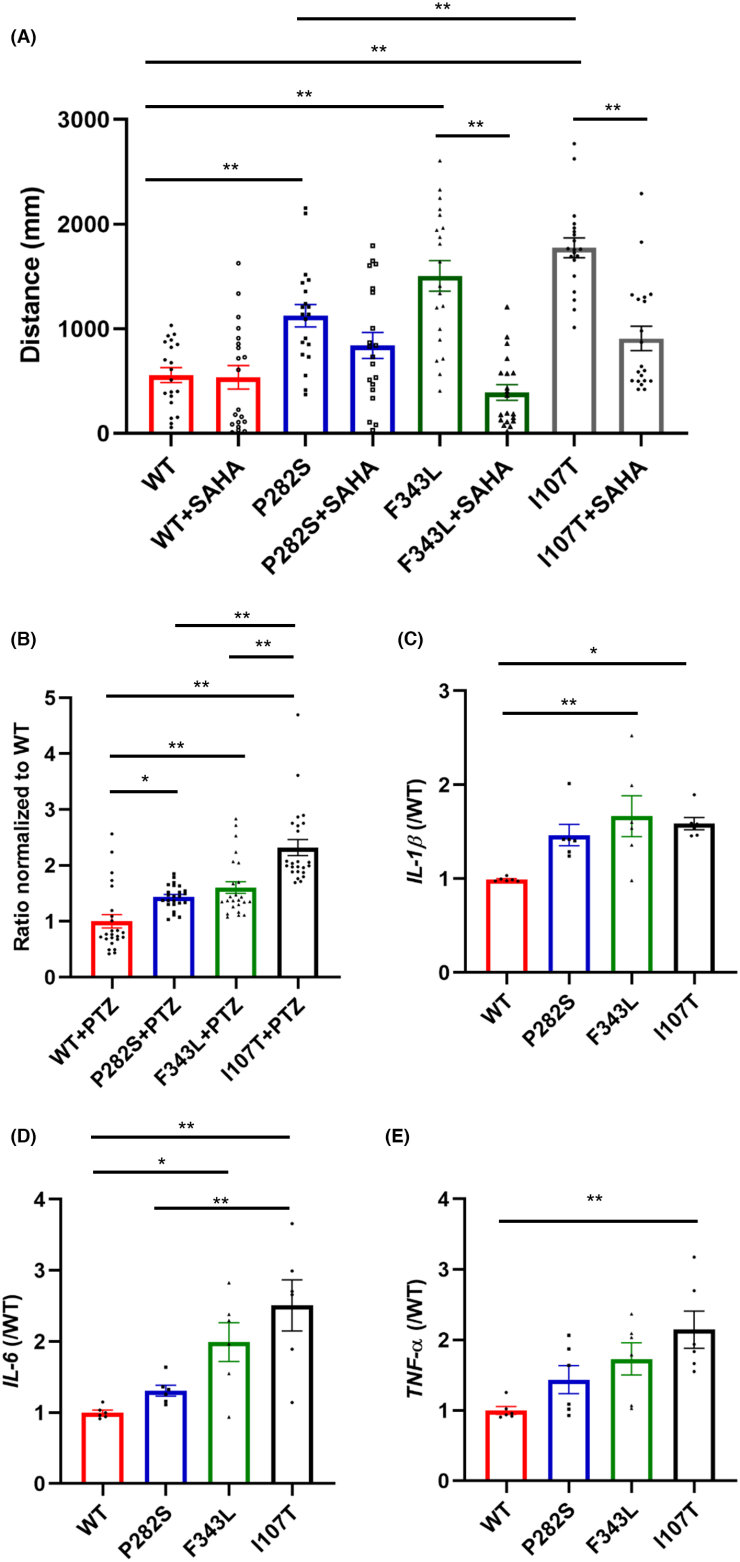
The comparison of behavioral activity, production of pro‐inflammatory factors, and response to SAHA treatment in different transgenic zebrafish. (A) The total distance of the P282S, F343L, and I107T transgenic zebrafish larvae traveled with 2.5 μM SAHA treatment. **p < 0.01 by one‐way ANOVA and subsequent Tukey's multiple comparisons (*n* = 20 in each group). (B) The response of the P282S, F343L, and I107T transgenic zebrafish larvae with 15 mM PTZ exposure. *p < 0.05, **p < 0.01 by one‐way ANOVA and subsequent Tukey's multiple comparisons (*n* = 20 in each group). The total distance of WT zebrafish traveled with PTZ exposure was set as “1”. (C) The production of IL‐1β in the P282S, F343L, and I107T transgenic zebrafish larvae at 5 dpf. *p < 0.05, **p < 0.01 by one‐way ANOVA and subsequent Tukey's multiple comparisons (*n* = 6 in each group and one sample = 10 pooled larvae). (D) The production of IL‐6 in the P282S, F343L, and I107T transgenic zebrafish larvae at 5 dpf. *p < 0.05, **p < 0.01 by one‐way ANOVA and subsequent Tukey's multiple comparisons (*n* = 6 in each group and one sample = 10 pooled larvae). (E) The production of TNF‐α in the P282S, F343L, and I107T transgenic zebrafish larvae at 5 dpf. **p < 0.01 by one‐way ANOVA and subsequent Tukey's multiple comparisons (*n* = 6 in each group and one sample = 10 pooled larvae).

Several de novo *GABRG2* pathogenic variants, including P282S, F343L, and I107T, have been reported to induce epileptic encephalopathy in patients.^5^ I107T occurred in the N‐terminal domain, while P282S and F343L occurred in the TM1 and TM3 transmembrane domains, respectively. To detect the effect of locations of mutations on phenotypes, we compared the locomotive activity of different mutant *GABRG2* transgenic zebrafish. In the previous research, we generated F343L and I107T transgenic zebrafish with spontaneous seizures.^6^, ^10^ In this research, the distance traveled in 30 min at 5 dpf was compared in the above three transgenic lines and the data showed that all mutant zebrafish traveled more than the WT control group, showing an epileptic phenotype. Among the three mutant zebrafish models, the I107T line traveled the most while the P282S line traveled the least, and the difference between the two groups was significant (1774 ± 94.55 mm in the I107T group vs. 1125 ± 106.9 mm in the P282S group, *p* = 0.0010, Figure 4A), indicating a less active phenotype induced by P282S mutation. Previous research has found a broad‐spectrum histone deacetylase (HDAC) inhibitor, SAHA, as an effective treatment for F343L and I107T zebrafish. Then we tried to detect whether it was also effective in the P282S zebrafish. However, the data showed that SAHA did not reduce the travel distance in the P282S zebrafish (Figure 4A), further suggesting that the phenotypes were different in the three mutant transgenic zebrafish. Moreover, the sensitivity of I107T zebrafish with PTZ exposure was also higher than that in the F343L and P282S zebrafish (P282S: 1.436 ± 0.04, *p* < 0.0001 vs. I107T; F343L: 1.604 ± 0.11 *p* < 0.0001 vs. I107T; I107T: 2.320 ± 0.14; Figure 4B).

To investigate whether neuroinflammation is involved in the three mutant zebrafish and whether it is related to the phenotypes of epilepsy, the production of pro‐inflammatory factors, including IL‐1β, IL‐6, and TNF‐α, was measured in the three mutant zebrafish lines. The data showed that, in the I107T zebrafish, all above inflammatory factors increased compared with the WT group (IL‐1β: 1.59 ± 0.06, *p* = 0.0171; IL‐6: 2.51 ± 0.33, *p* = 0.0008; TNF‐α: 2.14 ± 0.24, *p* = 0.0037) while there was no significant change in the P282S zebrafish (IL‐1β: 1.46 ± 0.10, *p* = 0.0710; IL‐6: 1.31 ± 0.07, *p* = 0.7779; TNF‐α: 1.44 ± 0.18, *p* = 0.4454) (Figure 4C,E). In addition, only IL‐1β (1.67 ± 0.20, *p* = 0.0064) and IL‐6 (1.99 ± 0.25, *p* = 0.0294) increased in the F343L zebrafish (Figure 4C,D). These data indicated that the production of proinflammatory factors varied among different mutant zebrafish and might be related to the severity of epileptic phenotypes.

### 3.5 Transcriptome changes in the brain of mutant *GABRG2* transgenic zebrafish

**FIGURE 5:**
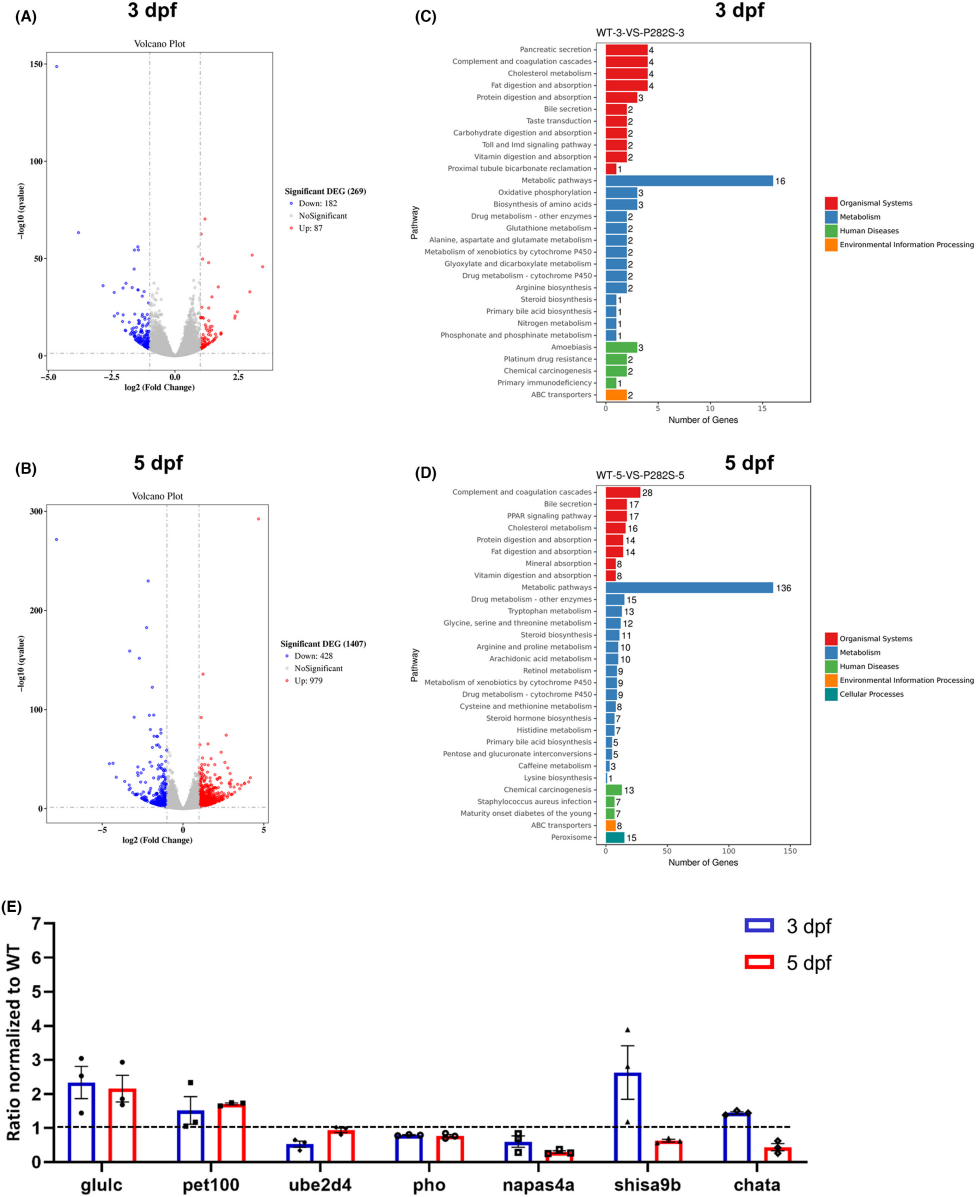
Brain transcriptional profile of Tg(*hGABRG2* ^*P282S*^) zebrafish larvae. (A) Volcano plot of differentially expressed genes (DEGs) in the brains of P282S zebrafish and the WT controls at 3 dpf. (B) Volcano plot of DEGs in the brains of P282S zebrafish and the WT controls at 5 dpf. (C) KEGG pathway of the DEGs at 3 dpf. (D) KEGG pathway of the DEGs at 5 dpf. (E) qPCR validation of the relative expression of DEGs in the P282S zebrafish. The expression level in the WT group was set as “1” after normalization to the internal reference gene *β‐actin* at 3 dpf and 5 dpf, respectively. *p < 0.05, **p < 0.01 by one‐way ANOVA and subsequent Tukey's multiple comparisons (*n* = 3 for each group and one sample = 10 pooled larvae). The full list of DEGs at 3 dpf and 5 dpf is provided in Tables S2 and S3.

To further investigate the mechanisms involved in P282S mutation‐induced epilepsy, brain transcriptome was analyzed. At 3 dpf and 5 dpf, the brain tissues of WT and P282S zebrafish were collected for transcriptome sequencing. There was 269 (87 down‐regulated and 182 up‐regulated) and 1407 (428 down‐regulated and 979 up‐regulated) differentially expressed genes (DEGs) in P282S group compared with the WT group at 3 dpf (Figure 5A, Table S2) and 5 dpf (Figure 5B, Table S3), respectively.

The KEGG pathway analysis showed that the number of DEGs related to metabolic pathways increased from 16 at 3 dpf to 136 at 5 dpf (Figure 5C,D). Among the DEGs at 5 dpf, 17 genes were related to peroxisome proliferator‐activated receptors (PPARs), 14 genes were related to protein digestion and absorption, 14 genes were related to lipid digestion and absorption, and 15 genes were related to drug metabolism (Figure 5D). We validated the expression of some DEGs by qRT‐PCR and the data showed that glutamate‐ammonia ligase (glutamine synthase) c (*glulc*) and PET100 homolog (*pet100*) increased at both 3 dpf and 5 dpf, ubiquitin‐conjugating enzyme E2D 4 (*ube2d4*), phoenix (*pho*), and neuronal PAS domain protein 4a (*npas4a*) decreased at both 3 dpf and 5 dpf, while shisa family member 9b (*shisa9b*) and choline O‐acetyltransferase a (*chata*) increased at 3 dpf and decreased at 5 dpf (Figure 5E). The result was consistent with the transcriptome analysis.

**FIGURE 6:**
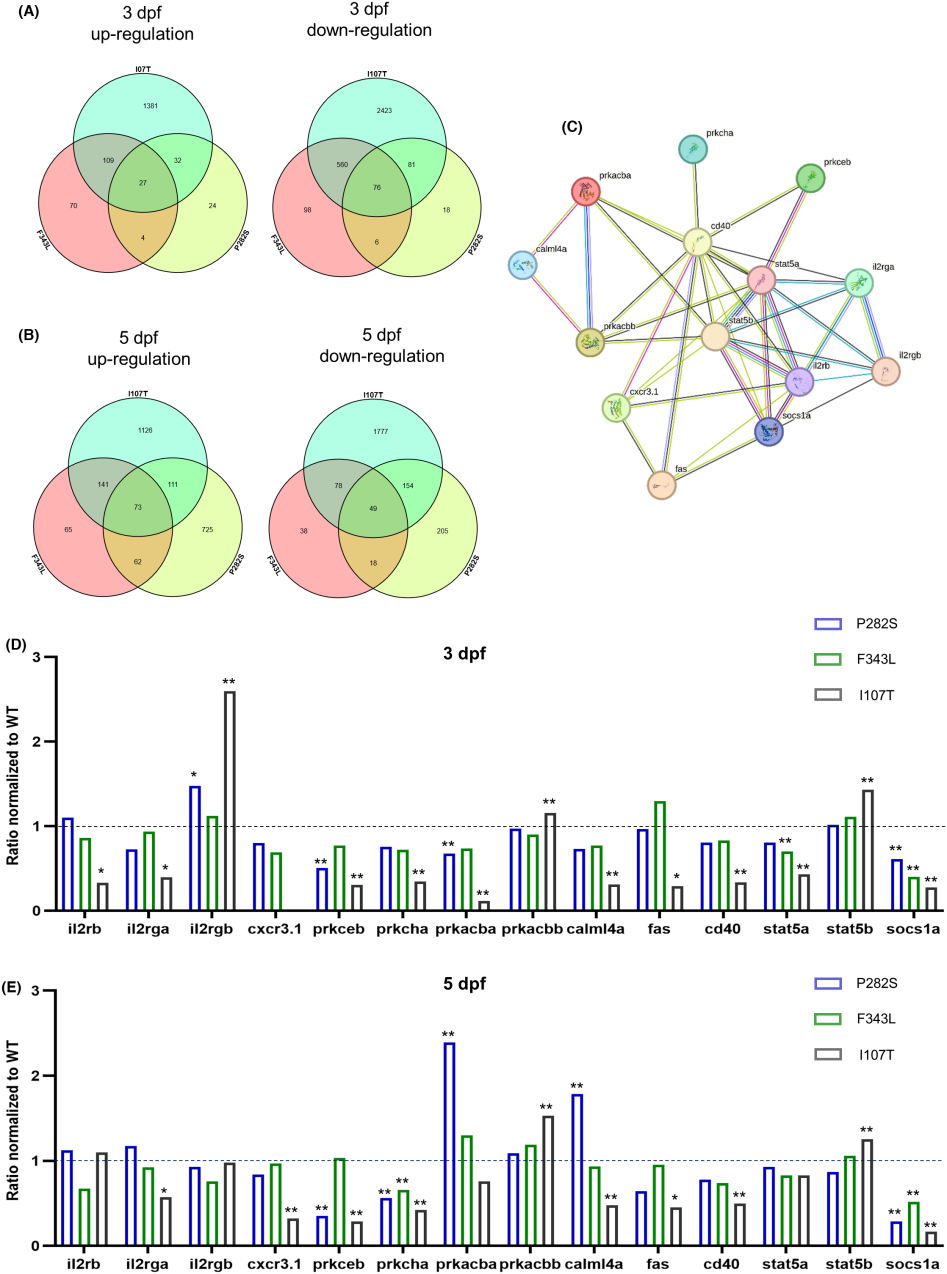
Differentially expressed genes in different mutant *GABRG2* transgenic zebrafish. (A) Venn diagram of the up‐regulated genes and down‐regulated genes in the brains of P282S, F343L, and I107T transgenic zebrafish larvae at 3 dpf. (B) Venn‐diagram of the up‐regulated genes and down‐regulated genes in the brains of P282S, F343L, and I107T transgenic zebrafish larvae at 5 dpf. (C) PPI network of DEGs at both 3 dpf and 5 dpf. (D) The relative expressions of DEGs in the PPI network in the P282S, F343L, and I107T transgenic zebrafish larvae at 3 dpf. (E) The relative expressions of DEGs in the PPI network in the P282S, F343L, and I107T transgenic zebrafish larvae at 5 dpf.

Lastly, the difference in the brain transcriptome of the three mutant zebrafish lines was compared. A total of 27 genes were upregulated and 76 genes were down‐regulated in all three lines at 3 dpf (Figure 6A), while 73 genes were up‐regulated and 49 genes were down‐regulated at 5 dpf (Figure 6B). The PPI network of the DEGs at both 3 dpf and 5 dpf was established with the STRING database, showing the involvement of interleukin‐2 (IL‐2) receptor signal pathway in the differential phenotypes of epilepsy caused by *GABRG2* mutations (Figure 6C). The expressions of DEGs in the PPI network, including IL‐2 receptor subunit beta (*il2rb*), IL‐2 receptor subunit gamma a (*il2rga*), IL‐2 receptor subunit gamma b (*il2rgb*), chemokine (C‐X‐C motif) receptor 3.1 (*cxcr3.1*), protein kinase C epsilon b (*prkceb*), protein kinase C eta a (*prkcha*), protein kinase cAMP‐dependent catalytic beta a (*prkacba*), protein kinase cAMP‐dependent catalytic beta b (*prkacbb*), calmodulin‐like 4a (*calml4a*), fas cell surface death receptor (*fas*), *cd40*, signal transducer and activator of transcription 5a (*stat5a*), signal transducer and activator of transcription 5b (*stat5b*), and suppressor of cytokine signaling 1a (*socs1a*), were compared in the three transgenic lines at 3 dpf (Figure 6D) and 5 dpf (Figure 6E). Among the three transgenic zebrafish lines, the expressions of *il2rb, il2rga, cxcr3.1, prkceb, prkcha, prkacba, calml4a, fas, cd40, stat5a* and *socs1a* were the lowest in the I107T zebrafish, while *il2rgb, prkacba* and *stat5b* were the highest, especially at 3 dpf. On the other side, the changes of genes in the IL‐2 receptor signal pathway in the P282S zebrafish were not as significant as that in the I107T zebrafish. The above data suggested the differential activation of the IL‐2 receptor signal pathway might be related to variable phenotypes of epilepsy.

## 4 DISCUSSION

*GABRG2(P282S)* missense mutation was first detected in a patient with epileptic encephalopathy, who had secondary generalized seizure onset at 1 year old and followed by atypical absences.^5^ Although the symptoms showed a slight improvement with lamotrigine treatment, the seizures remained intractable even after combination therapy with AEDs.^5^ To further investigate the potential mechanisms of genetic epilepsy induced by *GABRG2* mutations, we established the transgenic zebrafish line carrying the *GABRG2(P282S)* mutation in vivo. We also compared different phenotypes and analyzed the brain transcriptomic changes of the epilepsy models induced by different *GABRG2* mutations. Our data indicated that differential inflammation responses might determine the variable phenotypes of epilepsy induced by *GABRG2* mutations.

In the previous research, we established zebrafish epilepsy models by expressing mutant human *GABRG2(F343L)* and *GABRG2(I107T)*, which were discovered in patients. In this study, we constructed another epileptic zebrafish line with mutant *GABRG2(P282S)*, exhibiting a similar phenotype in humans. The successful establishment of these animal models provided a reliable approach for studying the pathological mechanisms of epileptogenesis and exploring drug treatment.

Some traditional AEDs, including CZP, LEV, CBZ, and VPA, were applied in the zebrafish model to find effective treatment for the patient. As CZP and LTG are both voltage‐gated sodium channel blockers,^12^, ^13^ the ineffectiveness of CZP and LTG in animal models and patients indicated that sodium channels were not significantly affected in the epilepsy induced by *GABRG2* mutations. LEV is one of the most prescribed broad‐spectrum AEDs, which has multiple effects, including inhibiting sodium channels, increasing the GABAergic transmission, and binding to SV2A to modulate the neurotransmitter release.^14^, ^15^, ^16^ Despite multiple molecular targets and mechanisms, the treatment with LEV did not show improvement of seizure activity in the mutant *GABRG2(P282S)* zebrafish model, further confirming the intractable phenotypes. On the other hand, CBZ and VPA showed a dose‐dependent effectiveness in the *GABRG2(P282S)* zebrafish, which was not effective in the *GABRG2(F343L)* zebrafish. CBZ is one of the broad‐spectrum AEDs against both generalized and focal seizures, which binds to GABA_A_ receptors and influences the opening of chloride channel.^12^ Although CBZ is mainly used as an add‐on treatment, it has been proven to be effective when used in monotherapy.^17^ VPA, one of the first line AEDs, which primarily enhances inhibitory neurotransmitter GABA,^13^ also significantly reduced the activity of *GABRG2(P282S)* zebrafish. The different actions of these AEDs in the *GABRG2(F343L)* and *GABRG2(P282S)* zebrafish indicated the different mechanisms or severity of these transgenic models. Not similar to *GABRG2(F343L)* and *GABRG2(I107T)* zebrafish, the HDACs inhibitor SAHA did not rescue the epileptic phenotype of *GABRG2(P282S)* zebrafish, further confirming the different phenotypes induced by various *GABRG2* mutations. Moreover, the different sensitivity to epileptic stimuli also demonstrated the hypothesis.

The VPA and SAHA are both broad‐spectrum HDACs inhibitors that target class I and IIa HDACs to increase the histone H3 acetylation level in the brain.^18^, ^19^, ^20^ In this study, they showed varied effects in the *GABRG2(P282S)* zebrafish. Although VPA has been used in clinics for decades, the mechanism remains elusive. The multipotential mechanisms of VPA include blocking T‐type calcium channels,^21^ enhancing the expression of glutamic acid decarboxylase, promoting the release of GABA from presynaptic terminals, inhibiting GABA degradation and GABA transaminase, and increasing GABA synthesis.^22^ From this point of view, we speculated that the regulation of the GABAergic pathway might be an important mechanism involved in the pharmacological effect of VPA in the *GABRG2(P282S)* zebrafish. However, whether the modulation of protein acetylation is involved in the pharmacological effect of VPA needs further investigation.

Our previous research has found increased inflammatory factors in mutant *GABRG2* transgenic zebrafish.^8^ Proinflammatory factors, such as IL‐1β, IL‐6, TNF‐α, and TGF‐β, were reported to play important roles in epileptogenesis,^23^, ^24^, ^25^ indicating that inflammatory response in the brain is a crucial mechanism in the pathophysiology of epilepsy.^26^ The activation of inflammatory response might destroy the balance between glutamate and GABA, leading to the increase of excitability in the nervous system and seizure activity.^27^ Here, we detected that the increase of pro‐inflammatory factors was related to the epilepsy severity, more pro‐inflammatory factors were found in the transgenic zebrafish showing more severe phenotype. The results suggested a positive correlation between inflammatory response and epileptic phenotype.

The comparison of brain transcriptome changes in the three zebrafish lines showed the differential activation of the IL‐2 receptor signal pathway. The role of IL‐2 in epilepsy is still controversial. The plasma level of IL‐2 was found to be lower,^28^ higher,^29^ or not significantly changed^30^ in epileptic patients than controls. However, these data were all collected from the patients after epileptic onset which could not exclude the influence of seizures on the expressions of cytokines. In our study, we detected differential expressions of IL‐2 receptor subunits at 3 dpf even before seizure onset, indicating that cytokine‐mediated inflammation might be one of the underlying mechanisms in the pathogenesis of epilepsy. IL‐2 signaling pathway was activated in patients with focal cortical dysplasia (FCD), a cause of intractable epilepsy, and JAK1/3‐STAT5 was the downstream of IL‐2‐dependent signaling pathways contributing to the pathogenesis of FCD.^31^ SOCS1 was shown to be able to interact with both JAK2A and STAT5.^32^ Here, we also observed differential expressions of *socs1, stat5a*, and *stat5b* in the three transgenic zebrafish, which was consistent with the changes in IL‐2 receptors. The results suggested that IL‐2 receptor activation might be involved in epileptogenesis through SOCS1/STAT5 signaling pathway. In addition, STAT5 was also regulated by IL‐1β and IL‐6.^33^ As different expressions of pro‐inflammatory factors and *stat5* expressions were found in the three transgenic zebrafish lines, we inferred that STAT5 signaling might be one of the important pathways responsible for epileptogenesis and be related to the severity of seizure onset.

Recently, the link of the immune system to mitochondrial dynamics and morphology was reported in two patients with severe neurological deterioration following viral infection, and JAK–STAT signaling might contribute to mitochondrial disease.^34^ As the brain is a highly energy‐demanding organ, metabolic dysfunction and mitochondrial defects contribute to acquired epilepsies, such as TLE.^35^ Metabolic dysfunction is crucial for epigenetics because the balance of neuronal activity requires bioenergetic adaptability.^36^ Although there was no direct evidence suggesting the metabolic dysfunction in genetic epilepsies, some researchers have suggested that metabolism and mitochondrial defects contributing to seizure susceptibility or progression in Dravet syndrome (DS) caused by *SCN1A* mutations and the ketogenic diet (KD) seemed to be an effective treatment for the patients.^37^, ^38^ In a zebrafish model of DS, the glycolytic and oxygen consumption rates were decreased.^39^ Here we observed dysregulation of the metabolic pathway in the P282S zebrafish, indicating the involvement of metabolic dysfunction in genetic epilepsy induced by *GABRG2* mutations. However, the relationship between inflammatory responses and metabolic changes needs further investigation.

## 5 CONCLUSIONS

In summary, we successfully established a transgenic zebrafish epileptic model expressing human mutant *GABRG2(P282S)*, which was sensitive to epileptic stimulus, and the metabolic pathway was significantly changed in the brains. CBZ and VPA showed antiepileptic effects in the transgenic zebrafish. Differential inflammatory responses, especially the SOCS/JAK/STAT signaling pathway, might be related to the phenotypes of genetic epilepsy induced by *GABRG2* mutations. Further study will expand the pathological mechanisms of genetic epilepsies and provide a theoretical basis for searching for effective drug treatment.

## AUTHOR CONTRIBUTIONS

ZQ and SD designed the research. SJ and ZL conducted experiments, analyzed the data, and drafted the manuscript. JS, WW, CY, YF, LW, and WJ assisted in zebrafish breeding, cell culture, and sample collection. ZQ, SD, and CM drafted and revised the manuscript. All authors contributed to the article and approved the submitted version.

## FUNDING INFORMATION

This work was supported by the National Natural Science Foundation of China (Grant No. 82371460, 82271487, and 81771404), the Natural Science Foundation of Jiangsu Province (Grant No. BK20201440), the Postgraduate Research & Practice Innovation Program of Jiangsu Province (KYCX23_3385, KYCX23_3413), and the Priority Academic Program Development of Jiangsu Higher Education Institutions (PAPD).

## CONFLICT OF INTEREST STATEMENT

The authors declare no conflicts of interest.

## CONSENT FOR PUBLICATION

All authors have read the manuscript and given their consent for publication.

## Supporting information


Figure S1.

Table S1.

Table S2.

Table S3.

## Data Availability

The raw data supporting the conclusions of this article will be made available by the authors.
