# Supplementary figures and images for: Differential inflammation responses determine the variable phenotypes of epilepsy induced by GABRG2 mutations
Source: CNS Neurosci Ther. 2024 Feb 15;30(2):e14583. doi: 10.1111/cns.14583 (PMC10867793; doi:10.1111/cns.14583)

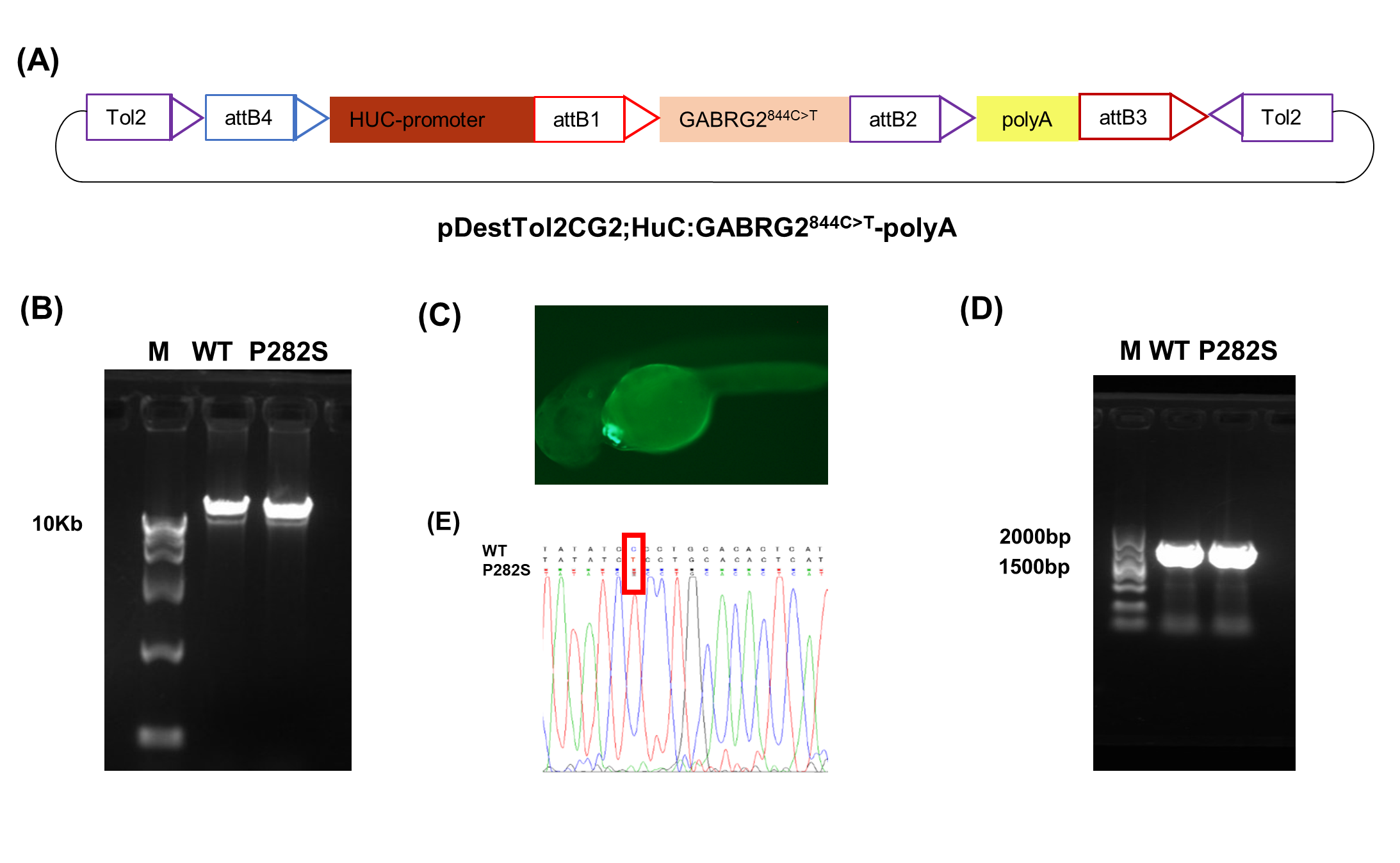

Supplement: Supplementary file 1 — Figure S1. [file CNS-30-e14583-s004.TIF]
